# Ophelia-like Paraneoplastic Limbic Encephalitis with Haemorrhagic Temporal Lobe Involvement and Cauda Equina Dysfunction in Classical Hodgkin Lymphoma: A Case Report

**DOI:** 10.3390/hematolrep18050064

**Published:** 2026-09-02

**Authors:** Abhishek Singla, Ritu Amit Chhabria, Michał Kurlapski, Michał Taszner, Jan Maciej Zaucha

**Affiliations:** Department of Hematology and Transplantology, University Clinical Centre, Medical University of Gdańsk, Smoluchowskiego 17, 80-211 Gdańsk, Poland

**Keywords:** Ophelia syndrome, classical Hodgkin lymphoma, paraneoplastic neurological syndrome, limbic encephalitis, mGluR5, negative neuronal antibody panel, case report

## Abstract

Ophelia syndrome is a rare paraneoplastic limbic encephalitis associated with classical Hodgkin lymphoma (cHL), most often with antibodies against metabotropic glutamate receptor 5 (mGluR5). We describe a 19-year-old man with newly diagnosed cHL who presented with generalised seizures, cognitive dysfunction, spastic paraparesis, cauda equina-related autonomic dysfunction, and a 35 × 31 mm haemorrhagic inflammatory lesion in the right temporal lobe. Brain biopsy showed dense intravascular and perivascular inflammatory infiltrates without neoplastic cells. Cerebrospinal fluid demonstrated pleocytosis and intrathecal IgG synthesis with type III oligoclonal bands. Serum and cerebrospinal fluid neuronal autoantibody panels were negative, but mGluR5 antibodies were not assessed. Cervical lymph node biopsy confirmed nodular sclerosis cHL, stage IIA. After exclusion of infectious encephalitis and central nervous system lymphoma, the presentation was considered most consistent with Ophelia-like paraneoplastic limbic encephalitis. ABVD chemotherapy was initiated, with rapid neurological improvement after the first cycle. Complete metabolic response was achieved after two cycles and sustained after six cycles. At 15-month follow-up, major neurological symptoms had not recurred, although bladder and bowel dysfunction persisted. This case highlights the importance of considering paraneoplastic limbic encephalitis in cHL despite negative standard neuronal antibody testing and of documenting whether mGluR5 antibodies were assessed.

## 1. Introduction

Ophelia syndrome is a rare paraneoplastic neurological syndrome most often linked to classical Hodgkin lymphoma (cHL). Only a limited number of cases have been reported, and the phenotype and optimal management remain incompletely defined [1]. In its classical form, the syndrome presents as limbic encephalitis with prominent neuropsychiatric symptoms and antibodies directed against metabotropic glutamate receptor 5 (mGluR5) [2].

Neurological complications in cHL are uncommon. When they occur, they are usually caused by direct compression or mass effect from lymphomatous lesions rather than by immune-mediated disease [3]. Direct central nervous system (CNS) involvement by cHL is exceptional, with an estimated incidence of approximately 0.02% [4]. Paraneoplastic neurological syndromes therefore become particularly relevant when seizures, behavioural change, or cognitive decline precede or accompany the diagnosis of lymphoma [5].

We describe a 19-year-old man with cHL who presented with seizures, a haemorrhagic inflammatory lesion in the right temporal lobe, cauda equina dysfunction, and rapid neurological improvement after lymphoma-directed chemotherapy, despite a negative standard serum and cerebrospinal fluid (CSF) neuronal antibody panel. This case supports the concept that an Ophelia-like paraneoplastic limbic encephalitis should not be dismissed solely because standard antibody assays are unrevealing, while also underscoring the need to state clearly when mGluR5 has not been assessed.

## 2. Case Presentation

A 19-year-old man was admitted after two generalised epileptic seizures. His previous medical history was notable only for childhood growth hormone deficiency. Subtle cognitive symptoms, primarily worsening concentration, had developed two months before admission. Neurological examination revealed spastic paraparesis, impaired bladder and bowel control, and deficits in attention and memory.

**Figure 1:**
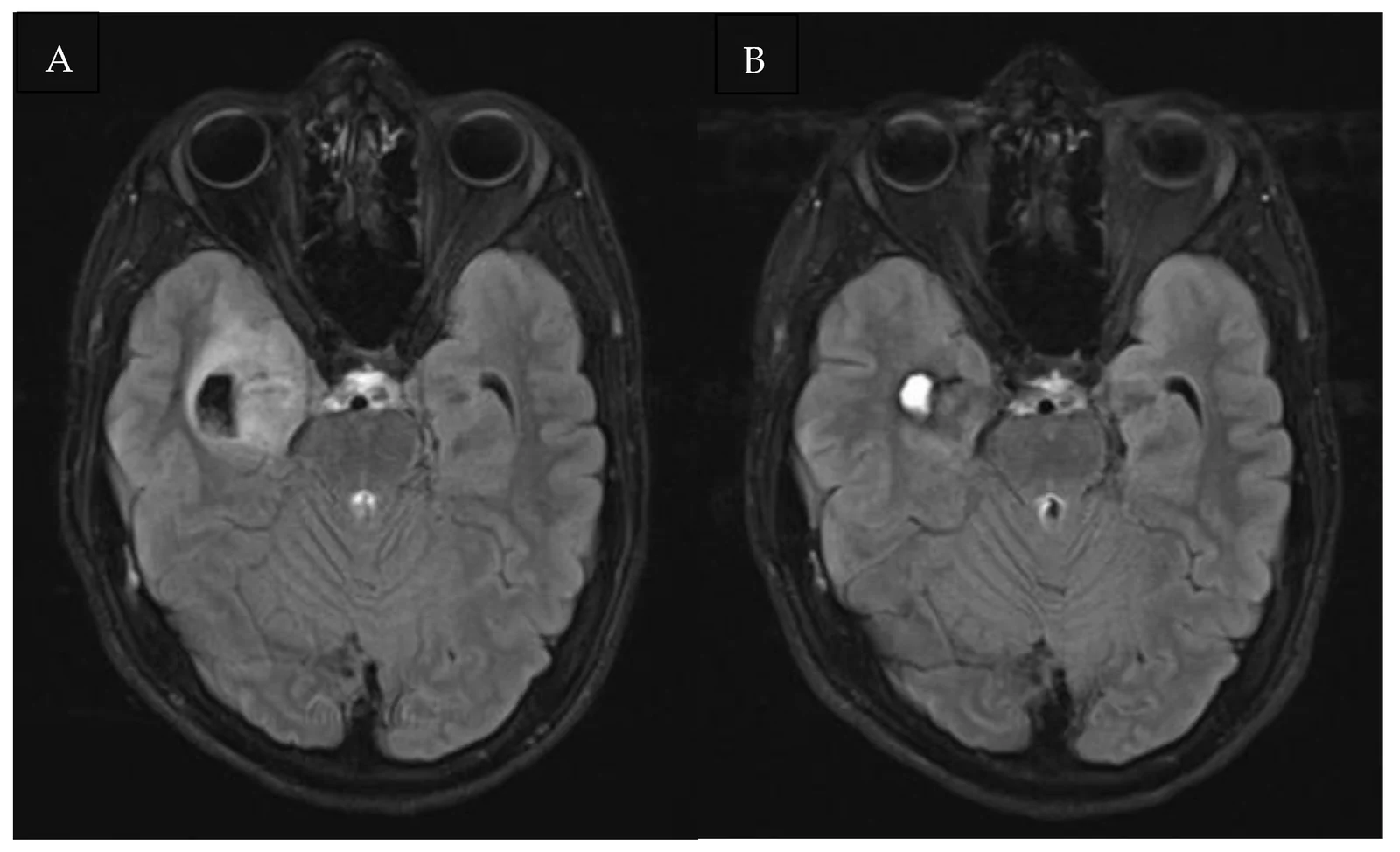
(**A**) Axial T2-weighted brain MRI obtained before stereotactic biopsy and cHL diagnosis, showing a right temporal lobe hyperintense lesion with haemorrhagic components. (**B**) Follow-up brain MRI performed after two cycles of ABVD during early response assessment.

**Figure 2:**
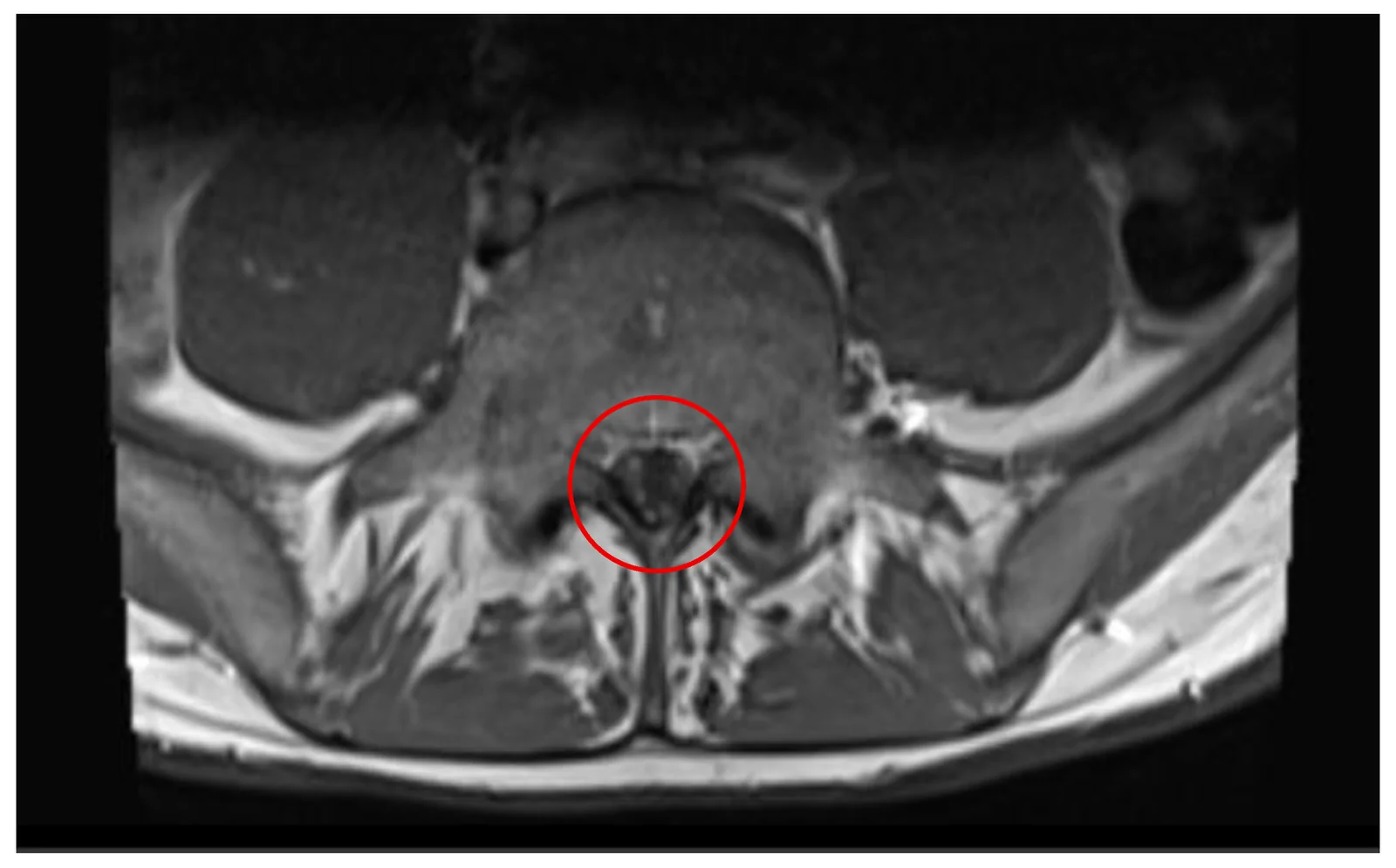
Axial contrast-enhanced T1-weighted spinal MRI showing enhancement of the cauda equina nerve roots (encircled).

Brain magnetic resonance imaging (MRI) demonstrated a focal right temporal lobe lesion measuring 35 × 31 mm on axial sections. The lesion was hyperintense on T2-weighted images and contained intralesional haemorrhage (Figure 1A). Because the imaging appearance raised concern for neoplastic, inflammatory, and vascular aetiologies, stereotactic biopsy was performed. Histology showed dense intravascular and perivascular inflammatory infiltrates without neoplastic cells. Contrast-enhanced spinal MRI demonstrated enhancement of the cauda equina nerve roots, supporting inflammatory involvement of the lumbosacral nerve roots (Figure 2).

CSF analysis showed pleocytosis (417 cells/µL; 89% lymphocytes, 9% monocytes, and 2% neutrophils), elevated total protein (2.1 g/L) and albumin (1.3 g/L) concentrations, and a markedly increased CSF IgG level (243.30 mg/L; reference < 34 mg/L). Flow cytometry showed no clonal cells. Serum IgG was 8.99 g/L (reference 6.1–16.16 g/L); oligoclonal IgG bands were detected in both serum and CSF. Additional CSF-restricted bands were present, corresponding to type III oligoclonal bands and supporting intrathecal IgG synthesis. Infectious studies, comprising bacterial and fungal testing and a broad-spectrum viral PCR panel, were negative. EBV DNAemia was negative, whereas EBV serology was not performed. A serum and CSF neuronal autoantibody panel including NMDA receptor, AMPA receptor, DPPX, CASPR2, LGI1, and GABA receptor antibodies was negative; mGluR5 antibodies were not included in the panel. Lupus anticoagulant was detected, whereas no other tested antiphospholipid antibodies were identified. ANA, anti-dsDNA, ENA, and nuclear and myositis immunoblot panels were negative. Nerve conduction studies showed normal motor conduction in the bilateral tibial and peroneal nerves and normal sensory conduction in the bilateral sural and superficial peroneal nerves.

At that point, the patient received intravenous methylprednisolone (1 g/day for 5 days), without clinical improvement.

Physical examination concurrently disclosed enlarged cervical lymph nodes. Excisional lymph node biopsy confirmed cHL, nodular sclerosis subtype. Immunohistochemistry showed CD15 positivity, CD30 positivity, CD45 negativity, PAX5 positivity, MUM1 positivity, Epstein–Barr virus-encoded RNA (EBER) positivity, and variable CD20 expression. Staging with positron emission tomography/computed tomography (PET/CT) classified the lymphoma as clinical stage IIA.

The working diagnosis required careful exclusion of several alternatives, particularly CNS lymphoma, infectious encephalitis, vasculitis, and other inflammatory lesions. The combination of seizures, limbic involvement with haemorrhagic features, cauda equina abnormalities, autonomic dysfunction, and systemic inflammatory activity, together with negative infectious testing and no radiological or histological evidence of CNS lymphoma, favoured a paraneoplastic neurological syndrome. Although mGluR5 antibodies were not assessed, the overall phenotype was most compatible with Ophelia-like paraneoplastic limbic encephalitis associated with cHL.

Given the progressive neurological decline and the presumed paraneoplastic mechanism, treatment was directed primarily at the lymphoma. ABVD (doxorubicin, bleomycin, vinblastine, and dacarbazine) chemotherapy was initiated approximately one month after admission. Neurological improvement was evident after the first cycle: paraparesis resolved and cognitive function improved, although bladder and bowel dysfunction persisted.

Treatment was complicated by episodes of agranulocytosis and transient grade 2 peripheral neuropathy. Vinblastine was therefore reduced by 50%, and treatment was continued as AVD (doxorubicin, vinblastine, and dacarbazine) from the third cycle onward. Interim PET/CT after two cycles showed a Deauville score of 1, consistent with complete metabolic response. At the same early response assessment, brain MRI demonstrated resolution of the previously observed temporal lobe lesion, with only residual post-haemorrhagic changes (Figure 1B). Despite the early complete metabolic response, treatment was continued for a total of six cycles as an individualised decision, given the unusually severe paraneoplastic neurological presentation and persistent neurological deficits, with the aim of maximising lymphoma control and potential neurological recovery. The patient ultimately received ABVD × 2 followed by AVD × 4. At the latest follow-up (15 months after treatment initiation), he remained in sustained remission without recurrence of major neurological symptoms. Persistent bladder and bowel dysfunction was considered likely to reflect residual cauda equina injury.

## 3. Discussion

Ophelia syndrome is generally understood as an immune-mediated complication of cHL, with mGluR5 acting as the best-characterised target antigen. mGluR5 is expressed in hippocampal and extralimbic regions, a distribution that helps explain the combination of amnesia, behavioural change, seizures, and other neuropsychiatric symptoms described in affected patients. The term “Ophelia syndrome” was introduced by Carr in 1982 to describe limbic encephalitis associated with Hodgkin lymphoma; nearly three decades later, Lancaster et al. identified antibodies against mGluR5 in two patients with this syndrome [2,6].

Most published patients have presented with mood or behavioural disturbance, anterograde amnesia, disorientation, seizures, or movement disorders. MRI abnormalities are reported in only about half of cases and usually appear as fluid-attenuated inversion recovery (FLAIR) hyperintensities in the medial temporal lobes; CSF often shows inflammatory changes such as pleocytosis [7,8]. Our patient shared the central limbic phenotype but differed in three clinically important ways: the temporal lobe lesion was haemorrhagic, the available neuronal antibody panel was negative while mGluR5 status remained unknown, and cauda equina dysfunction with persistent bladder and bowel involvement dominated the residual disability. The mechanism of haemorrhage remains uncertain; however, the prominent intravascular and perivascular inflammatory infiltrates may have compromised local vascular integrity and increased vessel fragility, predisposing to intralesional haemorrhage. No definite histological evidence of vasculitis was identified. These features suggest that the clinical spectrum of Ophelia-like syndromes associated with cHL may be broader than the antibody-positive cases reported to date.

Current diagnostic frameworks for autoimmune encephalitis and paraneoplastic neurological syndromes emphasise clinical context rather than antibody status alone [9,10]. Autoantibody testing should be performed in both serum and CSF, but negative results do not exclude autoimmune encephalitis. This is especially relevant in paraneoplastic presentations, where antibody titres may fall below assay thresholds, the relevant antigen may not yet be characterised, or tissue-restricted immune mechanisms may predominate. In the present case, mGluR5 was not tested, so the negative panel cannot be interpreted as true mGluR5 seronegativity. Conversely, isolated serum antibody positivity can be misleading; CSF findings and objective evidence of CNS inflammation are more specific when interpreted in the appropriate clinical setting [11].

This distinction was crucial in the present case. The diagnosis of an Ophelia-like syndrome with a negative standard neuronal antibody panel rested on converging evidence: seizures and memory impairment consistent with limbic encephalitis, an inflammatory CNS biopsy, intrathecal IgG synthesis, exclusion of infectious and metabolic causes, the absence of histological evidence of CNS lymphoma, the close temporal association with newly diagnosed cHL, and rapid improvement after lymphoma-directed therapy. Taken together, the findings made a paraneoplastic mechanism more persuasive than non-paraneoplastic seronegative autoimmune encephalitis.

Paraneoplastic syndromes in Hodgkin lymphoma may precede the lymphoma diagnosis and can affect the nervous, dermatological, haematological, endocrine, and rheumatological systems [12]. The neurological spectrum includes paraneoplastic cerebellar degeneration, limbic encephalitis including Ophelia syndrome, and granulomatous angiitis of the CNS [3,13,14]. Recognition of these syndromes is clinically important because neurological improvement may follow effective tumour control, as occurred in this patient.

The role of additional immunotherapy in cHL-associated paraneoplastic encephalitis remains uncertain, as responses to corticosteroids, IVIG, plasma exchange, and rituximab have been heterogeneous in published cases. In our patient, high-dose intravenous methylprednisolone did not result in clinical improvement, whereas neurological recovery became evident after lymphoma-directed chemotherapy. Although it cannot be excluded that IVIG or rituximab might have modified the neurological course, there is insufficient evidence to determine whether these therapies could have prevented the persistent autonomic deficits. We speculate that the residual bladder and bowel dysfunction reflected irreversible cauda equina and/or autonomic fibre injury established before effective control of the paraneoplastic process.

Table 1 summarises previously published cases of Hodgkin lymphoma-associated limbic encephalitis (Ophelia syndrome) with documented neuronal antibody status and compares them with the current case.

## 4. Limitations

This report has several limitations. mGluR5 antibodies were not assessed because the available neuronal antibody panel did not include mGluR5, and no stored CSF or serum samples were available for retrospective testing. Causality cannot be proven in a single case, and the improvement after chemotherapy could not be separated from the natural course of the inflammatory process. In addition, pathological confirmation was limited to the temporal lobe biopsy and did not establish the mechanism of cauda equina injury. These limitations should temper the use of the term Ophelia syndrome; for this reason, we describe the presentation as Ophelia-like rather than as a classical antibody-confirmed syndrome.

## 5. Conclusions

In patients with new-onset unexplained seizures, cognitive change, and inflammatory limbic lesions, paraneoplastic limbic encephalitis should remain in the differential diagnosis even when neuronal autoantibodies are negative. Concurrent lymphadenopathy should prompt urgent tissue diagnosis and staging. This case suggests that early lymphoma-directed therapy may contribute to neurological recovery in cHL-associated paraneoplastic neurological disease, whereas severe cauda equina involvement may result in persistent autonomic deficits. Comprehensive MRI, CSF analysis, neuropathological assessment when indicated, and systemic malignancy work-up are complementary in establishing the cause of neurological symptoms in patients with lymphoma. Early recognition of a possible paraneoplastic association may facilitate timely lymphoma-directed treatment and improve clinical outcomes.

## Figures and Tables

**Table 1:** Published reports and series of Hodgkin lymphoma-associated limbic encephalitis (Ophelia syndrome) with documented neuronal antibody status, compared with the current case. Abbreviations: ABVD, doxorubicin, bleomycin, vinblastine, and dacarbazine; ANNA-1, antineuronal nuclear antibody type 1; AVD, doxorubicin, vinblastine, and dacarbazine; CBA, cell-based assay; cHL, classical Hodgkin lymphoma; COPDAC, cyclophosphamide, vincristine, prednisone, and dacarbazine; CSF, cerebrospinal fluid; EuroNet-PHL, European Network for Paediatric Hodgkin Lymphoma; IVIG, intravenous immunoglobulin; IVMP, intravenous methylprednisolone; mGluR5, metabotropic glutamate receptor 5; NMDAR, N-methyl-D-aspartate receptor; OEPA, vincristine, etoposide, prednisone, and doxorubicin; PCA-2, Purkinje cell cytoplasmic antibody type 2; PET, positron emission tomography; PLEX, plasma exchange; PNMA2/Ta, paraneoplastic Ma antigen 2/Ta; SOX1, SRY-box transcription factor 1.

Author/Year	No. of Patients with cHL	Neuronal Antibody Status	Main Neurological Features	Treatment	Key Findings/Outcome
Hentschke et al., 2008 [15]	1	Anti-Hu (ANNA-1) positive	Limbic encephalitis with cognitive and memory impairment	ABVD	Near-complete neurological recovery
Zandi et al., 2009 [5]	1	Anti-NMDAR positive in serum and CSF	Memory impairment; limbic encephalitis associated with relapsed cHL	high-dose oral corticosteroids, IVIG, PLEX (10 days); gemcitabine/cisplatin	Neurological improvement after PLEX; progressive cHL
Lancaster et al., 2011 [2]	2	Anti-mGluR5 positive in serum; CSF not available for antibody testing	Memory loss, behavioural/psychiatric symptoms, seizures	Patient 1: ABVD + IVMP; Patient 2: mediastinal radiotherapy	Complete neurological recovery in both patients
Laffon et al., 2012 [16]	1	Anti-Hu positive in serum; negative in CSF	Confusion, memory loss, personality changes; frontal cognitive dysfunction	ABVD × 4 cycles	Spontaneous neurological improvement; complete recovery at 10 months; cHL remission
Mat et al., 2013 [7]	1	Anti-mGluR5 positive in CSF	Progressive memory impairment, personality/behavioural changes; lower cranial nerve palsies	ABVD × 6 cycles	Near-complete neurological recovery; complete cHL remission
Juneja et al., 2015 [17]	1	Serum anti-NMDAR negative; other neuronal/onconeural antibodies not tested	Temporal-lobe seizures, anxiety, hallucinations, short-term memory loss, weakness and autonomic dysfunction	IVMP, IVIG; ongoing ABVD	Progressive neurological deterioration; death after 4 weeks
Kunstreich et al., 2017 [18]	1	Anti-SOX1 positive in serum and CSF initially; PCA-2 positive in serum and CSF 7 months later	Cognitive impairment, disorientation; later spastic paraparesis and autonomic dysfunction.	IV corticosteroids, IVIG, PLEX, cyclophosphamide, azathioprine and rituximab; OEPA × 2 + COPDAC × 2 + mediastinal radiotherapy	Recurrent neurological relapses; persistent spastic paresis, neurogenic bladder and polyneuropathy
Spatola et al., 2018 [8]	5	Anti-mGluR5 positive	Psychiatric and cognitive symptoms; seizures	Chemotherapy in all (ABVD documented in 2/5); steroids in 3/5, PLEX in 1/5, IVIG in 1/5, radiotherapy in 1/5	Complete recovery in 4/5; residual moderate memory impairment in 1/5 Includes three previously reported cases (Lancaster et al. [2], *n* = 2; Mat et al. [7], *n* = 1)
Guevara et al., 2018 [19]	1	Anti-mGluR5 positive in CSF	Disorientation, agitation, hallucinations and anterograde memory impairment	IVMP; ABVD	Rapid neurological recovery
Petkov et al., 2020 [20]	1	Anti-Ma2/Ta (PNMA2/Ta) positive in serum; anti-mGluR5 not tested	Memory loss, confusion, hallucinations, opsoclonus–myoclonus and severe ataxia	IVMP; ABVD	Rapid neurological recovery
Kecskés et al., 2023 [21]	1	Anti-mGluR5 positive in serum	Right hemifacial allodynia, mild memory impairment and disorientation	ABVD, high-dose corticosteroids, rituximab, PLEX	Partial neurological recovery
Schnell et al., 2023 [22]	1	Anti-mGluR5 positive in serum and CSF	Acute psychosis, severe encephalopathy and autonomic dysregulation	IVMP, IVIG, immunoadsorption, rituximab and bortezomib, EuroNet-PHL protocol after cHL diagnosis	Complete neurological recovery; cHL diagnosed 16 months later with complete remission
Sanpei et al., 2023 [23]	1	Anti-mGluR5 negative in serum and CSF; broad neuronal antibody testing negative	Memory loss, impaired consciousness, seizures and myoclonus	IVMP; no cHL-directed therapy	Transient improvement followed by deterioration and death; cHL diagnosed at autopsy.
Pedrosa et al., 2024 [24]	1	Commercial CBA negative; anti-mGluR5 positive in serum and CSF by tissue-based assay and specific CBA	Visual hallucinations, memory impairment, mutism and nystagmus	IVMP, IVIG	Partial neurological improvement; cHL diagnosed 6 months later
Kumar et al., 2026 [25]	1	Autoimmune encephalitis panel negative; anti-mGluR5 status not reported	Behavioural changes, cognitive impairment and seizures	ABVD × 3 followed by AVD × 4	Neurological outcome not reported; cerebral PET abnormalities persisted at end of treatment.
Current case	1	Standard neuronal antibody panel negative in serum and CSF; anti-mGluR5 not assessed	Generalised seizures, cognitive dysfunction, spastic paraparesis and autonomic dysfunction; haemorrhagic temporal lesion	IVMP, ABVD × 2 followed by AVD × 4	Rapid neurological improvement after the first ABVD cycle; sustained cHL remission at 15 months; persistent bladder and bowel dysfunction

## Data Availability

All data generated or analysed during this study are included in this published article. Additional anonymised clinical information is available from the corresponding author on reasonable request, subject to patient confidentiality and institutional regulations.
